# EGHB010, a Standardized Extract of Paeoniae Radix and Glycyrrhizae Radix, Inhibits VEGF-Induced Tube Formation In Vitro and Retinal Vascular Leakage and Choroidal Neovascularization In Vivo

**DOI:** 10.1155/2017/1568702

**Published:** 2017-10-04

**Authors:** Eunsoo Jung, Wookwon Jung, Su-Bin Park, Chan-Sik Kim, Jin Sook Kim, Junghyun Kim

**Affiliations:** ^1^Laboratory of Toxicology, Research Institute for Veterinary Science and College of Veterinary Medicine, Seoul National University, Seoul, Republic of Korea; ^2^Department of Oral Pathology, School of Dentistry, Chonbuk National University, Jeonju 54896, Republic of Korea; ^3^Korean Medicine Convergence Research Division, Korea Institute of Oriental Medicine, Daejeon, Republic of Korea

## Abstract

EGHB010 is a hot water extract of the rhizome mixture of* Paeonia lactiflora* Pallas and* Glycyrrhiza uralensis* Fisch. Choroidal neovascularization (CNV) and vascular leakage are the common pathophysiologies of age-related macular degeneration. In this study, we aimed to evaluate the effect of EGHB010 on retinal vascular leakage and laser-induced CNV in a rat model. Vascular endothelial growth factor- (VEGF-) induced tube formation was assayed in human retinal microvascular endothelial cells. Intravitreal VEGF-induced blood-retinal barrier breakdown was assayed in Sprague-Dawley rats. Experimental CNV was induced by laser photocoagulation in Brown Norway rats. EGHB010 (50 and 100 mg/kg/day) was administered orally for 10 days after laser photocoagulation. Choroidal flat mounts were prepared to measure the lesion size of CNV. Incubation of retinal vascular endothelial cells with EGHB010 (12.5 and 25 *μ*g/mL) resulted in the inhibition of VEGF-induced tube formation in a dose-dependent manner. VEGF-mediated retinal vascular leakage was blocked by the oral administration of EGHB010. The CNV area was significantly lower in EGHB010-treated rats than in vehicle-treated rats. These results suggest that EGHB010 is a potent antiangiogenic agent. Thus, the oral administration of EGHB010 may have a beneficial effect in the treatment of vascular leakage and CNV in patients with age-related macular degeneration.

## 1. Introduction

Age-related macular degeneration (AMD) is the most common cause of vision loss in individuals older than 65 years [1]. Choroidal neovascularization (CNV) is a severe complication of AMD. AMD is classified into dry and wet forms. The dry form of AMD is characterized by the loss of retinal pigment epithelial cells and photoreceptors. The hallmark of wet or neovascular form of AMD is the growth of new vessels into the retina [2]. These newly formed vessels grow from the choroid through Bruch's membrane and lead to the formation of CNV in the subretinal pigment epithelium space. Diabetic retinopathy is a common complication of diabetes mellitus. Increased retinal vascular permeability caused by the breakdown of the blood-retinal barrier (BRB) results in diabetic macular edema, which is a major cause of vision loss in diabetic patients [3].

Vascular endothelial growth factor (VEGF) is a well-known proangiogenic and vascular permeability factor and a key mediator in the pathogenesis of wet AMD and diabetic retinopathy [4, 5]. Recently, the use of VEGF antagonists to inhibit VEGF signaling pathway has successfully diminished the formation of CNV in several experimental animal models [6] and human subjects [7]. In numerous clinical trials, intravitreally injected anti-VEGF agents, such as bevacizumab, ranibizumab, and aflibercept, notably suppressed neovascularization and stabilized vision loss in patients with neovascular AMD [8–10] and improved retinal edema and vision in patients with diabetic macular edema [11]. However, the intravitreal injection of anti-VEGF agents poses the risk of drug-associated or postinjection-associated adverse events [12, 13]. Repeated intravitreal injection increased the incidence of ocular complications, including endophthalmitis, ocular inflammation, traumatic cataract, intraocular pressure elevation, retinal detachment, and vitreous hemorrhage [14]. Despite the benefits of intravitreal anti-VEGF drugs, interest in the use of oral drug candidates has been increasing [15–17].

Some natural and synthetic compounds have been proposed as antiangiogenic agents [18]. Generally, botanical products are often perceived as safe when compared to synthetic compounds. Therefore, there has been an increasing interest in the use of herbal products [19]. EGHB010 is a hot water extract of the rhizome mixture of* Paeonia lactiflora* Pallas and* Glycyrrhiza uralensis* Fisch. (ratio of 2 : 1), which is formulated based on a well-known traditional herbal formula Jakyakgamcho-tang (Shaoyao-gancao-tang in Chinese; Shakuyaku-kanzo-to in Japanese). This herbal formula has been used as an analgesic and antispasmodic agent [20]. However, the inhibitory effects of this herbal formula on neovascular AMD and diabetic retinopathy have not been reported. Therefore, in this study, we investigated the inhibitory effects of EGHB010 on subretinal neovascularization in a rat model of laser-induced CNV and VEGF-induced vascular leakage in a rat model of BRB breakdown. We also investigated the inhibitory effect of EGHB010 on the VEGF-induced tube formation of human retinal microvascular endothelial cells.

## 2. Materials and Methods

### 2.1. Preparation of EGHB010

Standardized EGHB010 was provided by EYEGENE Co. Ltd. (Seoul, Korea). Paeoniae radix and Glycyrrhizae radix were purchased from CK herb store (Boeun, Chungcheongbuk-do, Korea) and Gamcho Farming Association Corporation (Jecheon, Chungcheongbuk-do, Korea), respectively. For the preparation of EGHB010, 200 kg of Paeoniae radix and 100 kg of Glycyrrhizae radix were weighed accurately and mixed. Distilled water (3,000 L) was added to the mixed herbs and extracted at 90°C for 8 h. The extract solution was filtered and concentrated to obtain a 50 kg extract. The extract was then mixed with maltodextrin (120 kg) as a carrier and stirred to form an aqueous solution. Then, the resulting mixture was spray-dried and filtered through a 400-mesh sieve to give an extract powder of EGHB010 (140 kg). The contents of the major components in EGHB010 were determined by high-performance liquid chromatography (HPLC) analysis according to the previously reported method [21].

### 2.2. Cell Viability Assay

Cell viability was examined using an MTS assay kit (CellTiter 96 AQueous One Solution Cell Proliferation Assay, Promega, WI, USA). Human retinal microvascular endothelial cells (HRMECs, Cell Systems, WA, USA) were plated (1 × 10^4^ cells/well) in quadruplicate into 96-well plates containing different doses of EGHB010 (0, 1, 5, 10, 25, 50, and 100 *μ*g/mL). Cell viability was measured 24 h after incubation. The results of MTS assay were obtained by measuring absorbance using a microplate reader (Tecan, Männedorf, Switzerland) at 490 nm. All experiments were repeated three times.

### 2.3. Tube Formation Assay

Tissue culture plates (96 well) were coated with 400 *μ*L of growth factor reduced basement membrane matrix (Matrigel, Becton Dickinson, NJ, USA). Human retinal microvascular endothelial cells (HRMECs, Cell Systems, WA, USA) were seeded at a density of 1 × 10^6^ cells/well and treated with serum-free CSC complete medium (Cell Systems) containing recombinant human VEGF (20 ng/mL) and EGHB010 (0, 12.5, and 25 *μ*g/mL) or ranibizumab (50 *μ*g/mL), a positive control anti-VEGF agent, for 17 h at 37°C. Capillary-like tube structures formed by HRMECs on the Matrigel were photographed with a digital camera (DP71, Olympus, Tokyo, Japan). Tube formation was quantified by counting the branching points of capillary-like structures per visual field. The experiments were repeated three times independently.

### 2.4. Laser-Induced CNV in Rats

Eight-week-old male Brown Norway (BN) rats (SLC, Hamamatsu, Japan) were anesthetized with isoflurane, and the pupils were dilated with topical 0.5% tropicamide ophthalmic solution (Santen Pharmaceutical, Osaka, Japan). To induce CNV formation, four photocoagulation sites were generated using a diode laser (577 nm wavelength, 0.1 s duration, 100 *µ*m spot size, 150 mW intensity, Oculight Slx, IRIS medical, CA, USA) between the retinal vessels equidistant from the optic nerve head in each eye. The formation of a subretinal bubble soon after laser treatment indicates the rupture of Bruch's membrane and induction of enough damage to form CNV. The rats were then divided into three groups of seven rats each as follows: (1) laser-induced CNV rats (CNV); (2) laser-induced CNV rats treated with EGHB010 (50 mg/kg body weight), and (3) laser-induced CNV rats treated with EGHB010 (100 mg/kg body weight). EGHB010 was dissolved in distilled water and orally administered for 10 days. The other group was given the same amount of vehicle gavage for 10 days. All procedures were approved by the Institutional Animal Care and Use Committee (IACUC Approval number 17-023).

### 2.5. Lectin Staining for CNV Size Analysis

At necropsy (10 days after laser treatment), all rats were sacrificed with CO_2_, and eyecups were fixed in 4% paraformaldehyde for 2 h. The eyecups were dissected to remove the anterior segment and neural retina. The RPE-choroidal complex was then stained with Rhodamine-labeled* Bandeiraea simplicifolia *isolectin B4 (BSI-IB4, Vector Laboratories, CA, USA). The CNV areas labeled with lectin were examined using a fluorescence microscope (Olympus, Tokyo, Japan). The size of CNV was calculated using the ImageJ software (NIH, MD, USA).

### 2.6. VEGF-Induced Retinal Vascular Hyperpermeability in Rats

VEGF-induced breakdown of the blood-retinal barrier (BRB) was induced according to the published protocol with modifications [22]. Briefly, seven-week-old male Sprague-Dawley rats were purchased from Koatech (Pyeongtaek, Korea). The rats were anesthetized with isoflurane and the pupils were dilated with 0.5% tropicamide (Santen Pharmaceutical, Osaka, Japan). Recombinant human VEGF (200 ng/1.0 *μ*L) was injected into the vitreous cavity of one eye using a microinjector (Hamilton, Reno, NV, USA). An equal volume of vehicle (PBS with 0.1% BSA) was injected into the other eye. The rats were then divided into three groups of seven rats each as follows: (1) intravitreal VEGF-injected rats (VEGF); (2) intravitreal VEGF-injected rats treated with EGHB010 (50 mg/kg body weight); and (3) intravitreal VEGF-injected rats treated with EGHB010 (100 mg/kg body weight). The rats were given EGHB010 (50 and 100 mg/kg) or vehicle gavage once daily for 3 days after the intraocular injection of VEGF. At the end of the study, rats were anesthetized by isoflurane inhalation. The fluorescein-dextran microscopy was performed according to a method described previously [23]. Briefly, fluorescein-dextran (Sigma, St. Louis, MO, USA) in phosphate buffered saline (PBS) at a concentration of 50 mg/mL was injected into the left ventricle. The tracer dye was allowed to perfuse for 15 min and the eyeballs were then placed in 4% paraformaldehyde for 1.5 h. The fluorescein-dextran perfused retinas were dissected and flat-mounted on a microscope slide. The whole-mount retinas were observed by fluorescence microscopy (Olympus, Tokyo, Japan). Plasma was collected from rat blood before sacrifice followed by centrifugation. The concentration of fluorescein-dextran in plasma was determined with a fluorometer (Synergy™ HT, Bio-Tek, VT, USA). The fluorescence intensity was determined by ImageJ software (National Institutes of Health, Bethesda, MD, USA) and normalized to the plasma fluorescein-dextran levels for each rat.

### 2.7. Statistical Analysis

Group data were analyzed by one-way analysis of variance followed by Tukey's multiple comparison test or unpaired Student's* t*-test using GraphPad Prism 6.0 software (GraphPad, San Diego, CA, USA). Differences with a *P* value < 0.05 were considered statistically significant.

## 3. Results

### 3.1. HPLC Analysis of EGHB010

The contents of the major compounds in EGHB010 were determined by HPLC analysis. Paeoniflorin (15.0%) and glycyrrhizin (5.1%) were found to be the major components of EGHB010 (Table 1).

### 3.2. EGHB010 Inhibits VEGF-Induced Tube Formation in HRMECs

**Figure 1:**
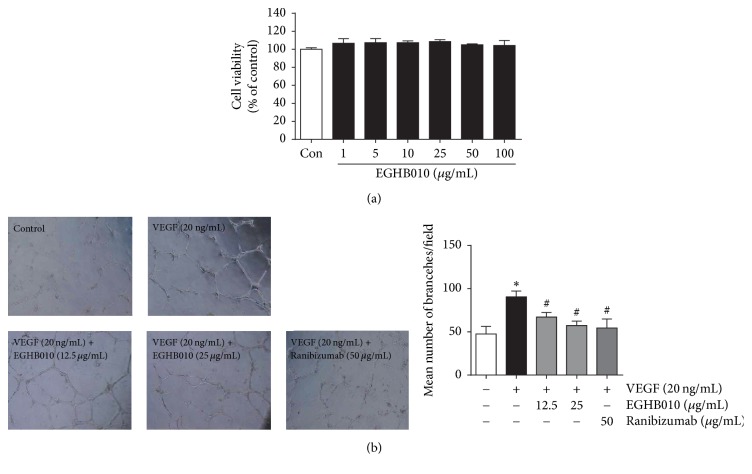
*EGHB010 inhibits tube formation in HRMECs*. (a) Effect of EHGB010 on the viability of HRMECs was determined by MTS assay. Data are expressed as percentage of control, *n* = 4. (b) HRMECs were treated with recombinant human VEGF (20 ng/mL) and EGHB010 (0, 12.5, and 25 *μ*g/mL) or ranibizumab (50 *μ*g/mL) for 17 h. Tube formation by HRMECs on Matrigel was observed with a microscope and quantified. Data are expressed as mean ± SEM, *n*  =  4. ^*∗*^*P* < 0.01 versus control; ^#^*P* < 0.01 versus VEGF.

To investigate the cytotoxic effect of EGHB010 on HRMECs, we performed MTT assay with various concentrations of EGHB010 (1–100 *μ*g/mL). The viability of EGHB010-treated HRMECs was not affected up to 100 *μ*g/mL (Figure 1(a)). Next, we examined whether EGHB010 could inhibit tube formation, an endothelial function crucial to angiogenesis, in HRMECs. VEGF was used as an angiogenic factor. HRMECs were significantly stimulated by VEGF and formed the capillary-like structures. However, EGHB010 treatment inhibited the formation of extensive capillary-like networks of HRMECs in a dose-dependent manner (Figure 1(b)). The inhibitory effect of EGHB010 on tube formation* in vitro* angiogenesis was similar to that of ranibizumab. These findings suggest that EGHB010 might inhibit VEGF-induced angiogenesis* in vitro*.

### 3.3. EGHB010 Inhibits VEGF-Induced Retinal Vascular Leakage

**Figure 2:**
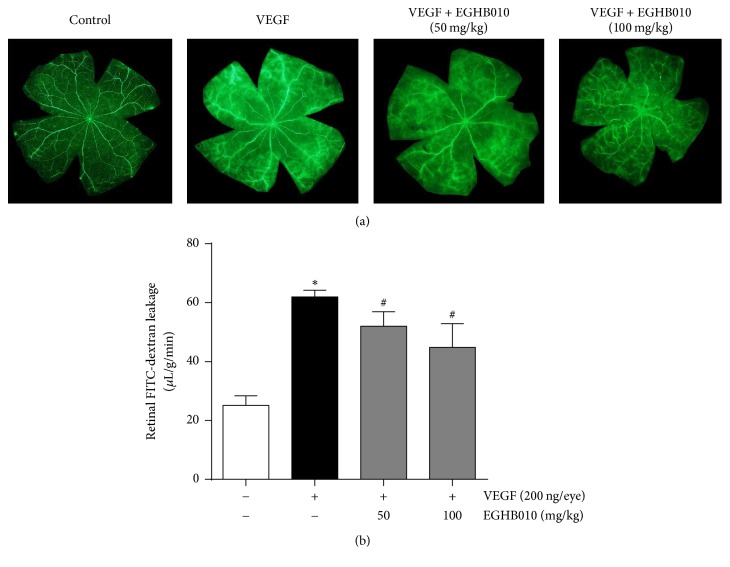
*EGHB010 inhibits VEGF-induced retinal vascular leakage*. (a) FITC-dextran angiography on retinal flat mounts. (b) Quantitative analysis of retinal vascular permeability. Values in the bar graphs represent the mean ± SEM, *n* = 7. ^*∗*^*P* < 0.05 versus control rats; ^#^*P* < 0.05 versus intravitreal VEGF-injected rats.

To examine VEGF-induced retinal vascular leakage* in vivo*, intravitreal injection of VEGF was administered and extravasation levels of fluorescein isothiocyanate- (FITC-) dextran were measured. The extravasation of tracer molecule was evident in rats treated with intravitreal VEGF. However, oral administration of EGHB010 ameliorated the angiographic features of VEGF-induced permeability (Figure 2).

### 3.4. EGHB010 Inhibits Laser-Induced CNV Formation

**Figure 3:**
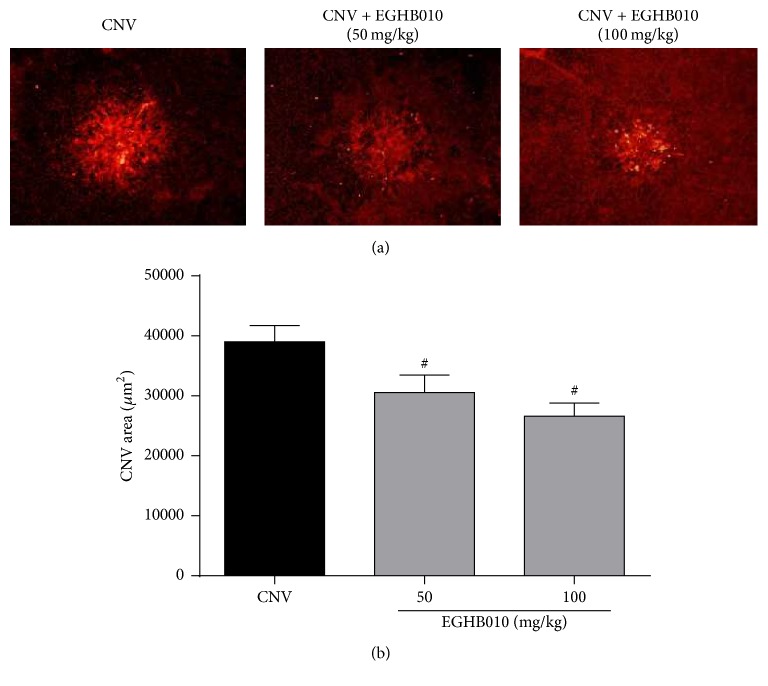
*EGHB010 inhibits laser-induced CNV formation*. (a) Choroidal flat mounts of laser-induced CNV. The CNV lesions were labeled with isolectin B4. (b) The areas of CNV lesions were measured in each group. The values in the bar graph represent the mean ± SEM, *n* = 7. ^#^*P* < 0.05 versus CNV rats.

The rats subjected to laser photocoagulation showed CNV formation at the laser photocoagulation site. This newly formed CNV was visualized by immunofluorescence staining with isolectin B4. Oral administration of EGHB010 significantly inhibited CNV formation in the subretinal areas (Figure 3(a)). As shown in Figure 3(b), the rats in the two groups treated with EGHB010 exhibited a reduction of 21.6 ± 7.6 and 31.8 ± 5.6% in CNV formation, respectively. This result showed that EGHB010 treatment significantly reduced the size of CNV, indicating that EGHB010 has potent antiangiogenic activity.

## 4. Discussion

Pathogenic angiogenesis and vascular leakage are the two main causes of severe vision loss in wet AMD and diabetic retinopathy [24]. VEGF and its receptors play an important role in the development of AMD and diabetic macular edema [4]. Inhibiting angiogenesis and vascular leakage by targeting VEGF has become a major focus of drug development for AMD and diabetic macular edema [25]. In the present study, we aimed to evaluate the effect of EGHB010 on retinal vascular leakage and laser-induced CNV in the rat models. We demonstrated for the first time that EGHB010 inhibited tube formation in HRMECs* in vitro* and retinal vascular leakage* in vivo,* mediated by VEGF. In addition, EGHB010 significantly suppressed CNV formation in a rat model of experimental laser-induced CNV. Taken together, our results suggest that the inhibitory effect of EGHB010 on vascular leakage and CNV formation is primarily via its potent anti-VEGF activity.

VEGF is a potent angiogenic and vascular permeability factor [26]. In the retina, elevated concentrations of VEGF correspond to BRB breakdown in animals [27] and humans [28]. Moreover, in neovascular AMD, the upregulation of proangiogenic molecules, such as VEGF, leads to the activation of angiogenic signal pathways and triggers CNV [29]. Numerous studies have suggested that VEGF plays a key role in retinal vasculopathy, and its inhibition significantly blocks the pathogenic alterations of retinal vasculature [30]. Recently, anti-VEGF agents have shown beneficial effects in patients with wet AMD [31].

Medicinal herbs are rich sources of potential preventive and therapeutic agents. EGHB010 is a standardized herbal extract, which is formulated based on a well-known traditional herbal medicine, and has an excellent safety record [32]. Our study for the first time showed the antiangiogenic effects of EGHB010* in vitro* and* in vivo*. Several studies have reported that some crude herbal extracts and phytochemicals can inhibit pathogenic neovascularization in tumorigenesis [33, 34] and retinal neovascular diseases [35–38]. In traditional Korean medicine, Paeoniae radix has been used to nourish blood, regulate menstruation, and alleviate pain. The extract of Paeoniae radix has been confirmed as the main therapeutic component of the medicinal herb for rheumatoid arthritis [39–41]. Rheumatoid arthritis is considered to be an angiogenic disease, because synovium is rich in newly formed vessels. The overexpression of VEGF has been demonstrated in the synovium of patients with rheumatoid arthritis [42]. Furthermore, the treatment of Paeoniae radix results in a reduction of neovascularization and a reduced expression of angiogenic factors [39, 41]. Recently, Deng et al. demonstrated a direct antiangiogenic effect of Paeoniae radix on human vascular endothelial cells and chick embryo chorioallantoic membrane [43]. Glycyrrhizae radix has been used to stop cough and detoxify several toxic substances. Kobayashi et al. showed that Glycyrrhizae radix inhibited tube formation of vascular endothelial cells* in vitro* and adjuvant-induced granuloma angiogenesis* in vivo* [44]. These findings support our observation that EGHB010 inhibited tube formation of HRMEC* in vitro* and experimental vascular leakage and choroidal neovascularization* in vivo*.

In many traditional herbal medicines containing a mixture of medicinal herbs, additive and synergistic effects of phytochemicals present in the different herbs have been observed. Notably, a multiherb formula occasionally possesses additive or synergistic effects, and the pharmacological activity, toxicity, and dosage are altered by the addition of other herbs [45]. EGHB010 has been used to treat muscle contraction and cramps. It was reported that this pharmacological effect of EGHB010 was only observed when Glycyrrhizae radix was mixed with Paeoniae radix [46]. EGHB010 contains two major compounds (paeoniflorin and glycyrrhizin). Paeoniflorin prevented oxidative stress-induced apoptosis in human RPE cells [47] and reduced VEGF levels in the synovium of rats with arthritis [48]. Glycyrrhizin inhibited neovascularization during tumor progression in mice [49]. Glycyrrhizin decreased VEGF production in rat retinal ganglion cells treated with advanced glycation end products [50]. In addition, glycyrrhizin has been known as a selective inhibitor of high-mobility group box-1, a potent proangiogenic molecule, and it attenuated ischemia-induced retinal neovascularization in a mouse model [51]. Although the detailed mechanism of action of EGHB010 as a VEGF inhibitor is still not clear, it is suggested that the antiangiogenic activity of EGHB010 may be due to the synergistic effects of paeoniflorin and glycyrrhizin.

In conclusion, this is the first study to provide evidence that EGHB010 inhibits experimental CNV formation and retinal vascular leakage. In addition,* in vitro* studies showed that EGHB010 treatment inhibited VEGF-induced tube formation in HRMECs. Further studies may be required to determine the feasibility of using EGHB010 in the treatment of human wet AMD and diabetic macular edema.

## Figures and Tables

**Table 1:** Paeoniflorin and glycyrrhizin content in EGHB010.

Compound	Content (mean ± SD, *n* = 3)
	mg/g
Paeoniflorin	15.00 ± 1.25
Glycyrrhizin	5.10 ± 0.20
